# Association of screen exposure with psychosocial problems in primary school students

**DOI:** 10.3389/fped.2022.961137

**Published:** 2023-01-13

**Authors:** Zedan Hu, Sitong Bi, Wei Wang, Chunjing Liu, Lihua Li

**Affiliations:** Children’s Center, Beijing Luhe Hospital, Capital Medical University, Beijing, China

**Keywords:** screen exposure, primary school students, psychosocial problems, family’s economic level, parental

## Abstract

**Introduction:**

This study aimed to investigate the current status of screen exposure and the factors related to screen exposure in primary school students and explore the relationship between screen exposure and psychosocial problems, which may provide evidence for the scientific use of electronic products and psychological interventions used in these children.

**Methods:**

The parents of 811 primary school students aged 6–12 years received a questionnaire survey in Beijing between January 13 and January 16, 2022. The demographic data and daily screen exposure time were collected, and the Strengths and Difficulties Questionnaire (SDQ) about their children was administered online.

**Results:**

In 793 students, the average daily screen exposure of <2 h and ≥2 h was noted in 75.0% and 25% of patients, respectively. The mobile phone was the main medium for screen exposure (40.9%). The family’s economic level, parental relationship, and main supervisor were related to screen exposure time (*χ*^2^ = 44.8,14.5 and 12.4, *P* < 0.05). A low family economic level with a monthly income not meeting the basic living needs, poor parental relationship, and an elderly person responsible for supervision were related to increased screen exposure time. The abnormal emotional and behavioral symptoms, conduct problems, hyperactivity inattention, peer communication, prosocial behaviors, and a total difficulties score were found in 11.6%, 9.8%, 15.3%, 22.1%, 6.8%, and 13.4% of children, respectively. Excessive screen exposure was related to peer interaction and prosocial behaviors (*χ*^2 ^= 12.18 and 7.76, *P* < 0.05). The children with excessive screen exposure were more likely to have abnormal peer interaction (*χ*^2 ^= 12.09, *P* = 0.001) and prosocial behaviors (*χ*^2 ^= 7.76, *P* = 0.005). Excessive screen exposure was a risk factor for peer interaction problems (*P* < 0.05).

**Discussion:**

In conclusion, the detection rate of excessive screen exposure is higher in primary school students, which is related to the family’s economic level, parental relationship, and main supervisor. Excessive screen exposure is harmful to the psychosocial health of these children, which is characterized by abnormal peer intercommunion and prosocial behaviors. More attention should be paid to screen exposure time in primary school students.

## 1. Introduction

In recent years, electronic devices (such as televisions, computers, mobile phones, and other electronic products) have become popular with the advancement of technology, and therefore, the activities of children related to electronic devices (screen exposure) are increasing. Studies have shown that excessive screen exposure in children and adolescents can not only cause poor vision, obesity, cardiovascular disease, sleep problems, and poor academic adaptability (1–4) but also affect their psychological and behavioral development (5–8). The psychological behavior problems in children and adolescents may progress into adulthood and have a negative influence on later mental and behavioral health (9). Previous studies have focused on the impact of screen exposure on the mental health of preschool children. This study aimed to investigate the current status of and factors affecting screen exposure in primary school students through a questionnaire survey and explore the relationship between screen exposure and the mental health of these children, which may provide evidence on the scientifical use of electronic products and interventions on the poor psychological behavior problems in primary school students.

## 2. Materials and methods

### 2.1. Subjects

An online questionnaire survey was administered to the parents of primary school students aged 6–12 years in Beijing between January 13, 2022, and January 16, 2022. Parents of 811 children received a questionnaire survey. Of them, eight refused to participate in this study, the response to the survey was shorter than 120 s for parents of three children, a history of epilepsy was noted for three children, a history of tic disorder was found for three children, and a history of brain trauma was found for one child. Finally, the questionnaires from parents of 793 children were subjected to further analysis. Informed consent was obtained before the study, and this study was approved by the Ethics Committee of Beijing Luhe Hospital, Capital Medical University (batch number: 2022-LHKY-017-03).

### 2.2. Methods

#### 2.2.1. Collection of general characteristics

The general characteristics of children and their parents were collected, including gender, age, grade, education level of parents, family type, family’s economic level, parental relationship, medical history of children, family history, the major supervisor in the family, and attitude of grandparents and parents to education.

#### 2.2.2. Screen exposure information

The mean daily screen exposure time was calculated for the semester from September 2021 to January 2022 (except for the screen exposure time for routine learning). The screen exposure time was divided into six levels: 0–30 min/d; 30 min–1 h/d; 1–2 h/d, 2–3 h/d, 3–4 h/d, and >4 h/d. According to the Guidance to the Physical Activity for Chinese Children and Adolescents, the daily screen exposure time should be shorter than 2 h (10). In the present study, the cutoff time was defined as 2 h. The daily screen exposure times of <2 h and ≥2 h were defined as normal and abnormal, respectively.

The Strengths and Difficulties Questionnaire (SDQ) was initially developed by an American psychologist, Goodman (11), and mainly employed as a measure of behavioral and emotional problems in children and adolescents. It has been widely used in the United States, Netherlands, Germany, and other countries. In 2005, the SDQ was modified by Kou et al., and its reliability and validity were tested in Shanghai norms (12). The questionnaire includes five scales: emotional symptoms scale, conduct problems scale, hyperactivity scale, peer problems scale, and prosocial scale, and there were a total of 25 items. Each item was graded 0–2 (0, not true; 1, somewhat true; 2, certainly true). The emotional symptoms were scored as follows: 0–3, normal; 4, borderline; 5–10, abnormal; the conduct problems were scored as follows: 0–2, normal; 3, borderline; 4–10, abnormal; hyperactivity was scored as follows: 0–5, normal; 6, borderline; 7–10, abnormal; peer problems were scored as follows: 0–2, normal; 3, borderline; 4–10, abnormal; prosocial behaviors were scored as follows: 6–10, normal; 5, borderline; 0–4, abnormal; total difficulties were scored as follows: 0–13, normal; 14–16, borderline; 17–40, abnormal. This questionnaire has been confirmed to possess favorable reliability with a Cronbach *α* coefficient of 0.784.

### 2.3. Quality control

The reasonability and suitability of this questionnaire were assessed in our pilot studies, and then, it was improved according to the findings from a pilot study. The questionnaire was administered to parents by the school. All items were scored, and the score of each item was recorded for further analysis. The parents of eight children refused to participate in this study; the response to the questionnaire survey was shorter than 120 s for the parents of three children, three children had a history of epilepsy, three had a history of tic disorder, and one had a history of brain trauma.

### 2.4. Statistical analysis

Statistical analysis was performed with SPSS version 25.0. Qualitative data were expressed as a frequency or percentage and compared with a *χ*^2^ test. A value of *P* < 0.05 was considered statistically significant. Multiple testing was taken into account using Bonferroni's correction (*α*′ = 0.017). A value of *P* < 0.017 was considered statistically significant. Logistic regression analysis was employed to investigate the factors influencing the screen exposure time to psychological behavior problems (normal, borderline, and abnormal) in primary school students, with a *P*-value < 0.05 considered statistically significant.

## 3. Results

### 3.1. Screen exposure and contributing factors

After excluding seven individuals with a past history of confirmed epilepsy (three individuals), convulsions (three individuals), and previous cranial trauma (one individual), patients of 793 children received a questionnaire survey, including 409 boys and 384 girls. No psychiatric disorders in three generations of close relatives of both parents' families were founded. The main electronic devices responsible for screen exposure were mobile phones (40.9%), TVs (31.8%), and iPads (23.6%). The daily screen exposure times of <2 h and ≥2 h were found in 75.0% and 25% of children, respectively. The daily screen exposure time was closely related to the family's economic level, parental relationship, and major supervisor in the family (*P* < 0.05) but had no relationship with gender, grade, education level of parents, type of family, and consistent attitude to education (*P* > 0.05) (Table 1). The factors with significant differences in the univariate analysis served as independent variables, and the screen exposure time served as a dependent variable (0, time of screen exposure <2 h; 1, time of screen exposure ≥2 h) in the logistic regression analysis. The results showed that a low family economic level with a monthly income not meeting the basic living needs, poor parental relationship, and grandparents as major supervisors increased the risk of screen exposure (OR = 0.50, 1.46, and 1.97, *P* < 0.05) (Table 2).

**Table 1:** Univariate analysis of screen exposure time in primary school students.

Variable		Daily screen exposure time (%)		*χ* ^2^	*P*
		<2 h	≥2 h		
Gender	Boy	298 (50.1)	111 (56.1)	2.12	0.15
	Girl	297 (49.9)	87 (43.9)		
Grade	1	101 (17.0)	31 (15.7)	5.96	0.31
	2	113 (19.0)	31 (15.7)		
	3	138 (23.2)	48 (24.2)		
	4	120 (20.2)	37 (18.7)		
	5	72 (12.1)	23 (11.6)		
	6	51 (8.6)	28 (14.1)		
Education level of the father	Junior high school or lower	80 (13.5)	37 (18.7)	4.93	0.30
	Senior high school	267 (44.9)	89 (45.0)		
	Junior college	174 (29.2)	46 (23.2)		
	Undergraduate degree	57 (9.6)	19 (9.6)		
	Higher than an undergraduate degree	17 (2.9)	7 (3.5)		
Education level of the mother	Junior high school or lower	77 (12.9)	33 (16.7)	6.79	0.15
	Senior high school	228 (38.3)	70 (35.4)		
	Junior college	190 (31.9)	61 (30.8)		
	Undergraduate degree	91 (15.3)	26 (13.1)		
	Higher than an undergraduate degree	9 (1.5)	8 (4.0)		
Family type	Original family	565 (95.0)	182 (91.9)	3.44	0.18
	One-parent family	19 (3.2)	8 (4.0)		
	Remarried family	11 (1.8)	8 (4.0)		
Family income per month	Difficult in basic requirements for life	10 (1.7)	25 (12.6)	44.8	<0.001
	Meeting basic requirements for life	320 (53.8)	107 (54.0)		
	Surplus in basic requirements for life	265 (44.5)	66 (33.3)		
Parental relationship	Harmonius	496 (83.4)	141 (71.2)	14.5	0.001
	General	48 (8.1)	31 (15.7)		
	Inharmonius	51 (8.6)	26 (13.1)		
Main supervisor	Father or mother	500 (84.0)	144 (16.0)	12.4	<0.001
	Grandparents	95 (16.0)	54 (27.3)		
Attitude to education	Highly consistent	84 (14.1)	32 (16.2)	0.51	0.77
	Basically consistent	432 (72.6)	141 (71.2)		
	Basically inconsistent	79 (13.3)	25 (12.6)		

**Table 2:** Logistic regression analysis of factors influencing the screen exposure time in primary school students.

Variables	Dependent variable	Regression coefficient	Standard error	Wald *χ*^2^	*P*	OR	OR (95% CI)
Family income per month	Time of screen exposure	−0.70	0.15	21.81	<0.001	0.50	0.37–0.67
Parental relationship		0.38	0.12	10.06	0.002	1.46	1.16–1.84
Main supervisor		0.68	0.19	12.17	<0.001	1.97	1.35–2.89

### 3.2. Analysis of psychological and behavioral status

In 793 children, the emotional symptoms, conduct problems, hyperactivity and inattention, abnormal peer interaction, prosocial behaviors, and abnormal total difficulties scores were observed in 11.6%, 9.8%, 15.3%, 22.1%, 6.8%, and 13.4% of the children, respectively. The detection rate of abnormal peer interaction was the highest (Table 3).

**Table 3:** Incidence of emotional and behavioral symptoms in primary school students.

Variables	Normal (*n* %)	Borderline (*n* %)	Abnormal (*n* %)
Emotional symptoms	624 (78.7)	77 (9.7)	92 (11.6)
Conduct symptoms	632 (79.7)	83 (10.5)	78 (9.8)
Hyperactivity and inattention	576 (72.6)	96 (12.1)	121 (15.3)
Abnormal peer interaction	474 (59.8)	144 (18.2)	175 (22.1)
Prosocial behaviors	659 (83.1)	80 (10.1)	54 (6.8)
Total difficulties score	595 (75.0)	92 (11.6)	106 (13.4)

### 3.3. Correlation of screen exposure time with SDQ

Results showed that screen exposure time was closely related to peer interaction and prosocial behaviors (*χ*^2 ^= 12.18 and 7.76, *P* < 0.05) (Table 4). Children with excessive screen exposure were more likely to have abnormal peer interaction (*χ*^2 ^= 12.09, *P* = 0.001 < 0.017) and abnormal prosocial behaviors (*χ*^2 ^= 7.76, *P* = 0.005 < 0.017).

**Table 4:** Correlation of screen exposure time with SDQ in primary school students.

Variables		Daily screen exposure time (%)		*χ* ^2^	*P*
		<2 h	≥2 h		
Emotional symptoms	Normal	476 (80.0)	148 (74.7)	2.63	0.27
	Borderline	53 (8.9)	24 (12.1)		
	Abnormal	66 (11.1)	26 (13.1)		
Conduct symptoms	Normal	483 (81.2)	149 (75.3)	3.90	0.14
	Borderline	60 (10.1)	23 (11.6)		
	Abnormal	52 (8.7)	26 (13.1)		
Hyperactivity and inattention	Normal	436 (73.3)	140 (70.7)	1.04	0.59
	Borderline	68 (11.4)	28 (14.1)		
	Abnormal	91 (15.3)	30 (15.2)		
Abnormal peer interaction	Normal	372 (62.5)	102 (51.5)	12.18	0.002
	Borderline	109 (18.3)	35 (17.7)		
	Abnormal	114 (19.2)	61 (30.8)		
Prosocial behaviors	Normal	503 (84.5)	156 (78.8)	7.76	0.02
	Borderline	60 (10.1)	20 (10.1)		
	Abnormal	32 (5.4)	22 (11.1)		
Total difficulties score	Normal	459 (77.1)	136 (68.7)	5.82	0.05
	Borderline	62 (10.4)	30 (15.2)		
	Abnormal	74 (12.4)	32 (16.2)		

### 3.4. The relationship between screen exposure time and psychological behavior problems in primary school students

We performed a logistic regression analysis with exposure time as the independent variable, while each factor of SDQ and the total number of difficulties were considered dependent variables (normal, edges, and abnormal). The results showed that excessive screen exposure was a risk factor for prosocial behavior and peer interaction problems in primary school students (all *P* values < 0.05). After adjusting for monthly family’s economic level, parental relationship, and major supervisor, excessive screen exposure was significantly related to peer problems (*P* < 0.05). Furthermore, after adjusting for gender, grade, education level of parents, family type, monthly family’s economic level, parental relationship, the major supervisor in the family, and attitude toward education, excessive screen exposure was still significantly associated with peer problems (Table 5).

**Table 5:** Relationship between screen exposure time and psychological behavior problems in primary school students.

Normal or edges vs. Abnormal	OR (95% CI)^a^	*P*	^a^OR (95% CI)^b^	*P*	^a^OR (95% CI)^c^	*P*
Emotional	1.2 (0.74–1.95)	0.45	1.19 (0.71–1.98)	0.51	1.22 (0.71–2.1)	0.46
Conduct	1.57 (0.95–2.59)	0.08	1.48 (0.86–2.56)	0.16	1.48 (0.84–2.6)	0.18
Hyperactivity	0.98 (0.63–1.54)	0.93	0.78 (0.48–1.27)	0.32	0.79 (0.48–1.31)	0.37
Peer problem	1.86 (1.29–2.68)	0.001	1.66 (1.13–2.43)	0.01	1.56 (1.05–2.34)	0.03
Prosocial behavior	2.18 (1.24–3.85)	0.007	1.59 (0.86–2.94)	0.14	1.65 (0.87–3.14)	0.13
Total difficulties scores	1.35 (0.86–2.11)	0.20	1.35 (0.84–2.18)	0.22	1.38 (0.83–2.29)	0.22

^a^ Screen exposure.

^b^ Adjusted for monthly family economic income, parental relationship, and major supervisor.

^c^ Adjusted for gender, grade, education level of parents, family type, monthly family economic level, parental relationship, major supervisor in the family, and attitude toward education.

## 4. Discussion

### 4.1. Current status of screen exposure in primary school students

Our study indicated that approximately 25% of children had a daily time of screen exposure no shorter than 2 h, which was higher than that reported in previous studies. Liu et al. (4) investigated screen exposure in 276 children aged 3–12 years, and their results showed more than 15% of children had a daily screen exposure time longer than 2 h during the COVID-19 global pandemic period. Ma et al. investigated the daily screen exposure time in children and adolescents aged 6–17 years from seven provinces in China, and they found that as high as 22.5% of participants had a daily screen exposure time longer than 2 h (13). These findings indicate that the daily screen exposure time is relatively long in primary school students, which may be explained by the COVID-19 global pandemic. Although COVID-19 remains stable globally, there are still local outbreaks in China. Thus, some children have reduced outdoor activities and gathering activities, aiming to reduce the risk of viral spread. Thus, time at home increases for these students, which therefore increases the risk of screen exposure. Of note, a Brazilian study revealed that the daily screen exposure time was longer than 2 h in 63% of preschool students (14). In another Chinese study, the proportion of adolescents with a daily screen exposure time longer than 2 h was about 41.5% in 12 provinces (15). The proportion in our study was lower than that in the above two studies, which might be ascribed to the exception of screen exposure time spent on learning. In addition, the differences in study design and age might also be related to the discrepancy in available studies. In addition, our study revealed that the mobile phone was the major medium for screen exposure, which was consistent with that reported by Wu et al. (15). Thus, it is necessary to limit the use of electronic devices, especially mobile phones, in primary school students.

### 4.2. Factors affecting daily screen exposure time

Our study indicated that screen exposure time was related to the family's economic level, parental relationship, and main supervisor in the family. Children in a family with lower income were more likely to have a longer screen exposure time. In a family with a low income, the parents may work longer to make a living and thus fail to pay enough attention to their children and emphasize the education of children (16). On the contrary, opposite findings have also been revealed in other studies. A study indicates that the screen exposure time of children in a family with high income is longer than that in a family with a low income (17). A harmonious parental relationship is a protective factor for screen exposure in primary school students. In a family with a harmonious parental relationship, the parents have more comprehensive scientific parenting knowledge and can accompany children patiently and considerately; the parent–child interaction increases, and therefore the supervision of children is enhanced, leading to reduced screen exposure time. Furthermore, when the grandparents were the main supervisor of students, the children were more likely to have a longer screen exposure time. Under this condition, the interaction between children and grandparents is reduced, and grandparents are more likely to spoil children, leading to the increased use of electronic devices. Therefore, for a family with low income, a disharmonious parental relationship, and grandparents as the main supervisor of children, more attention should be paid to the care and education of children.

### 4.3. Relationship between screen exposure and psychological health

Our study showed that 13.4% of primary school students had abnormal total difficulties scores, and the detection rate of abnormal peer interaction was high. This proportion was higher than that reported by Zeng et al. in a survey of school-aged children in Liuzhou City (11.8%) (18) and that reported by Luan et al. (19) in a survey of school-aged children in Shanghai (9.6%). This suggests that the detection rate of emotional and behavioral problems is high in primary school students, and abnormal peer interaction is a prominent problem. In our study, the relationships of screen exposure with five scales of emotional and behavioral problems and the total difficulties score were evaluated. Our results showed that screen exposure was closely related to peer interaction and prosocial behaviors. The students with excessive screen exposure were more likely to have abnormal peer interaction and prosocial behaviors than those with normal screen exposure. In the primary school stage, the extent of peer interaction is expanded and communication is more frequent. Prolonged screen exposure may inevitably reduce peer interaction among students. However, peer interaction is a key factor in maintaining good interpersonal relationships and integrating into society, which plays a vital role in the healthy psychological development of children. A Turkey study (20) showed that children with excessive screen exposure time had significantly higher scores for emotional symptoms, conduct problems, abnormal peer relationships, and total difficulties scores. A study from the United Kingdom (7) revealed that the daily time of watching TV, video, or DVDs no shorter than 3 h was associated with an increase in behavioral problems in children, but there were no close associations between screen exposure and abnormal peer interaction or prosocial behaviors. Some Chinese studies have also found that excessive screen exposure is related to emotional and behavioral problems in preschool children (21, 22). Zhang et al. conducted a study on 7,200 Chinese adolescents aged 13–18 years from six regions in China (23). Their results showed the detection rates of emotional symptoms, behavioral symptoms, difficulty in social adaptability, and psychological symptoms were high in the adolescents with screen exposure time >2 h/d. In addition, prolonged screen exposure increases the risk of prolonged sedentary time. Studies have shown that sedentariness is associated with poor mental health (24). There is evidence showing that prolonged screen exposure may increase social problems, mental problems, rule-breaking behaviors, and aggressive behaviors (25). The psychological problems in early childhood have implications for predicting mental health, psychosocial, and antisocial (including criminal) behaviors in early adulthood. If the psychological problems in childhood cannot be promptly treated and resolved, related symptoms may continue into the adolescent period or even adulthood, causing adverse effects on individuals, families, and society. Based on the results of the present study, some early emotional and behavioral problems of primary school students should be paid more attention to, and targeted measures should be taken in time to improve the psychological health of primary school students. We advocate that schools and families should pay attention to the mental health of elementary school students and reduce screen exposure time by increasing sports, outdoor activities, and interactive parent–child games. Parents are also encouraged to accompany children to watch screens and discuss screen contents together, which can also increase parent–child interactivity. More knowledge about mental health should also be disseminated to help parents and children to identify early psychological problems and adjust their psychological state in time to prevent the development of serious problems.

## 5. Conclusions

Taken together, screen exposure time is relatively long in primary school students, the family environment affects the screen exposure time, and screen exposure is related to the psychological and behavioral health of primary school students. Parents should appropriately reduce the time of screen exposure (especially mobile phones) in primary school students and increase their outdoor activities, which may benefit healthy mental development.

## Data Availability

The raw data supporting the conclusions of this article will be made available by the authors without undue reservation.
